# Bacterial Adhesion on Orthopedic Titanium Implants Coated with Silver-Doped Hydroxyapatite and Chitosan

**DOI:** 10.3390/molecules31193537

**Published:** 2026-10-04

**Authors:** Urška Filipović, Anamarija Zore, Nives Matijaković Mlinarić, Anže Abram, Roman Štrukelj, Martina Modic, Andreja Leskovac, Sandra Petrović, Vojislav Stanić, Safia Dahmen, Raja Gošnak Dahmane, Klemen Bohinc

**Affiliations:** 1 Faculty of Health Sciences, University of Ljubljana, 1000 Ljubljana, Slovenia; urska.filipovic@gmail.com (U.F.); anamarija.zore@zf.uni-lj.si (A.Z.); nmatijak@irb.hr (N.M.M.); roman.strukelj@zf.uni-lj.si (R.Š.); raja.dahmane@zf.uni-lj.si (R.G.D.); 2 University Medical Center Ljubljana, 1000 Ljubljana, Slovenia; 3 Jozef Stefan Institute, 1000 Ljubljana, Slovenia; anze.abram@ijs.si (A.A.); martina.modic@ijs.si (M.M.); 4 Vinča Institute of Nuclear Sciences—National Institute of the Republic of Serbia, University of Belgrade, 11351 Belgrade, Serbia; andreja@vin.bg.ac.rs (A.L.); sandra@vin.bg.ac.rs (S.P.); voyo@vin.bg.ac.rs (V.S.); 5 Laboratory of Transmissible Diseases and Biologically Active Substances LR99ES27, Faculty of Pharmacy, University of Monastir, Monastir 5000, Tunisia; safia_dah@yahoo.fr

**Keywords:** bacterial adhesion, titanium implants, chitosan, silver-doped hydroxyapatite

## Abstract

Orthopedic site infections remain a growing concern in implantology, particularly in light of rising antibiotic resistance. Identifying optimal, standardized antimicrobial treatments is increasingly challenging. Beyond the design and functional properties of orthopedic materials, key factors such as bacterial adhesion and genotoxicity must also be considered. To develop implant materials with enhanced antibacterial properties and biocompatibility, this study investigated titanium (Ti) surfaces coated with chitosan (CS) and silver-doped hydroxyapatite (AgHA) at weight ratios of 1:1, 1:2, and 2:1. The incorporation of silver ions into hydroxyapatite demonstrated potent antibacterial activity, achieving a greater than 99% reduction in *Staphylococcus aureus* (*S. aureus*) viability in solution after just two hours of incubation, effectively inhibiting further bacterial growth. Furthermore, CS coatings combined with AgHA significantly reduced *S. aureus* adhesion and proliferation on Ti surfaces, with over 96% reduction in total bacterial viability. Further biological evaluation was performed on the CS:AgHA = 2:1 coating, selected for its favorable surface and antibacterial characteristics. Genotoxicity and cytotoxicity assessments using human peripheral blood lymphocytes showed no significant increase in micronuclei incidence for either uncoated Ti or CS:AgHA = 2:1-coated surfaces. Additionally, cell viability remained above 84%, indicating favorable biocompatibility under the experimental conditions used. Overall, these findings highlight the potential of CS:AgHA = 2:1 composite coatings for further investigation in antibacterial orthopedic implant applications.

## 1. Introduction

Surgical site infections (SSIs) account for approximately 20% of healthcare-associated infections, with 2–5% of inpatient surgical patients affected. Many infections are discovered post-discharge, underestimating their true prevalence [1,2,3]. Orthopedic implant infections pose a significant global medical challenge, leading to prolonged hospital stays, increased morbidity and mortality, and high economic costs. Infection rates in primary joint replacement are below 1–2% but can rise to 40% after revision surgery. These infections are classified as early (within three months), delayed (3–24 months), or late (beyond 24 months). Given the growing prevalence of antibiotic-resistant bacteria, infection prevention strategies are crucial [4,5].

A multidisciplinary approach involving microbiologists, physicists, clinicians, and orthopedic surgeons is essential. The functional properties of orthopedic materials—mechanical strength, chemical stability, microstructure, and biocompatibility—play a critical role in their effectiveness. Although the development of new antimicrobial drugs has slowed, research into antimicrobial coatings for implants has intensified [6].

Although resistance to standard antibiotics is increasing, the development and approval of new antimicrobial drugs are decreasing [7]. In recent years, great scientific efforts have been made to explore various ways in which orthopedic implants can be antimicrobial, e.g., through the development of antimicrobial coatings.

Titanium (Ti) and its alloys are widely used in orthopedic implants because of their favorable mechanical properties, corrosion resistance, and biocompatibility. However, the native titanium surface lacks intrinsic antibacterial activity and may therefore support bacterial attachment and subsequent biofilm formation. Consequently, considerable attention has focused on surface-engineering strategies designed to modify the physicochemical and biological properties of titanium while preserving its favorable bulk properties [8,9]. These strategies include mechanical, physical, chemical, and electrochemical treatments that can modify surface topography, roughness, wettability, and surface chemistry, as well as the fabrication of micro- and nanoscale features that influence cellular and bacterial interactions with the implant surface [10,11,12,13].

From an antimicrobial perspective, surface modifications can be broadly designed to reduce bacterial attachment through passive mechanisms or to exert direct antimicrobial effects through active mechanisms. Passive approaches include modifying surface topography and wettability, as well as applying polymer brushes, hydrophilic layers, and textured surfaces that inhibit bacterial adhesion, whereas active strategies incorporate antimicrobial agents such as antibiotics, antimicrobial peptides, metal ions, or metallic nanoparticles that actively eliminate pathogens [11]. Composite coatings that combine these approaches are particularly attractive because they may simultaneously support osseointegration and limit bacterial colonization [12,13,14,15,16]. Although numerous methods for preparing composite coatings and modifying titanium surfaces have demonstrated promising results, critical questions regarding coating stability, long-term efficacy, and biological safety remain to be fully addressed.

Among polymeric materials investigated for titanium surface modification, chitosan (CS), a biopolymer derived from chitin, has attracted considerable interest because of its biocompatibility, biodegradability, and antimicrobial properties [17]. Its antimicrobial activity has been primarily associated with electrostatic interactions between protonated amino groups of CS and negatively charged bacterial cell surfaces, which can alter membrane integrity and interfere with bacterial growth. However, the limited solubility of CS at neutral and alkaline pH may restrict its application under physiological conditions, leading to the development of chemically modified CS derivatives with improved solubility and antimicrobial efficacy [18,19,20,21,22,23]. Studies have confirmed its firm fixation to titanium alloys, further improving antimicrobial potential [5,24,25].

Silver is another widely investigated antimicrobial component for biomedical applications owing to its oligodynamic properties. Its antimicrobial activity is associated with the release of silver ions and their interactions with bacterial membranes, proteins, and intracellular components. However, the biological effects of silver are concentration-dependent. While low concentrations are non-toxic to humans, excessive amounts can lead to tissue deposition and argyria. Generalized argyria has been reported in humans exposed to ionic and nanocrystalline silver at cumulative doses ranging from 70 to 1500 mg/kg of body weight [26].

Therefore, incorporation of silver into implant coatings requires careful consideration of both antimicrobial efficacy and potential cytotoxicity. One strategy for controlling silver exposure while maintaining antimicrobial activity is its incorporation into bioactive matrices such as hydroxyapatite. Silver-containing hydroxyapatite has demonstrated antibacterial activity against clinically relevant pathogens and has shown potential for reducing biofilm formation on implant-related surfaces [27,28].

Bacterial adhesion to implant surfaces is the first and most important step in infection and leads to the formation of biofilms in which the bacteria are more resistant to antimicrobial agents [29]. The pathogens that cause implant-related infections can be quite heterogeneous. The most common bacteria found in samples of infected tissue or on extracted implants are Gram-positive cocci: *Staphylococcus aureus* (*S. aureus*) and *Staphylococcus epidermidis* (*S. epidermidis*), Gram-negative bacteria: e.g., *Pseudomonas aeruginosa*, and mixed flora [7]. This level of microbial diversity shows that the microbiological identification of bacteria using resistance testing is essential. Empirical therapy without reliable prior identification of the bacteria must be avoided. Because surface characteristics can influence bacterial attachment, evaluating bacterial adhesion is an important component of assessing antimicrobial implant coatings.

Alongside antimicrobial performance, the biological safety of modified implant surfaces is a critical consideration in their development. Biocompatibility testing to evaluate the interaction between a product and tissue/bone is mandatory for the development and approval of orthopedic materials. According to the International Organization for Standardization (ISO) 10993-3 [30], biocompatibility evaluation of biomaterials includes several specific in vitro and in vivo tests, the application of which depends on the end use of the material [31].

Genotoxicity and cytotoxicity testing are essential parts of the safety evaluation of materials intended for close body contact or permanent implantation. It enables the identification of compounds that affect the genetic material of cells and cause DNA damage directly or indirectly through various mechanisms. In addition, cytotoxicity testing provides insight into the potential of a material or compound to impair cell viability, inhibit proliferation, or induce cell death, which are critical parameters when evaluating biocompatibility and safety.

The present study investigated Ti plates coated with different ratios of silver-doped hydroxyapatite (AgHA) and chitosan. The coatings were characterized in terms of surface roughness and contact-angle measurements. Their biological performance was further evaluated by examining bacterial adhesion using *S. aureus* and assessing genotoxicity and cytotoxicity. This approach was used to investigate whether CS-AgHA coatings can provide a combination of favorable surface characteristics, antibacterial performance, and acceptable biocompatibility for potential application to titanium implant surfaces.

## 2. Materials and Methods

### 2.1. Preparation of AgHA Nanoparticles and CS-AgHA Composites

CaCO_3_, Ag_2_O, H_3_PO_4_, and glycerol with a purity of p.a. (Merck/Sigma-Aldrich, Darmstadt, Germany) were used as starting materials. The CS used had an average molecular weight (190,000–310,000 Da) and a degree of deacetylation of 75–85% (Merck/Sigma-Aldrich, Darmstadt, Germany).

The synthesis of AgHA was prepared by the neutralization method in an air atmosphere [32]. Calcium oxide (obtained by calcination of CaCO_3_ at 1000 °C) was suspended in 500 mL of distilled water, which was heated to 95 °C. The solution of 300 mL H_3_PO_4_ (0.5 M) and Ag_2_O (0.0006 mole) was dropped into the Ca(OH)_2_ (0.24898 mole) suspension until the endpoint was reached (pH = 7). The resulting suspension was stirred at room temperature for about twenty hours. The precipitate obtained was filtered through a vacuum filtration process. The precipitate was washed with water, dried at 105 °C, and pulverized in a mortar.

The CS solution was prepared by dissolving CS (0.25 g) in 1% acetic acid (25 mL) with stirring for 1 h to obtain a transparent solution. In this study, commercially pure Ti was used as a substrate for coating CS-AgHA composites. It was cut into a 10 mm × 10 mm plates with a thickness of 0.2 mm. The plate was polished with 100 to 3000 grit SiC paper and ultrasonically cleaned in distilled water and ethanol. The aqueous dispersion of AgHA (25 mg in 10 mL H_2_O) and glycerol (0.075 mL) were added to the CS solution, stirred at room temperature for 30 min and then treated in an ultrasonic bath for 15 min. The weight ratio of CS:AgHA in the dispersion was 1:2, 1:1, and 2:1. Thin films of CS-AgHA dispersion were obtained by spin-coating 0.1 mL of the dispersion onto the Ti plate at a spin speed of 1500 rpm for 60 s. The obtained films were dried in a drying oven at 50 °C for 30 min and stored in a desiccator.

### 2.2. Characterization of the Synthesized CS-AgHA Composites

The FTIR spectra of the prepared material samples were recorded at ambient conditions in the mid-IR range (400–4000 cm^−1^ with a resolution of 4 cm^−1^) using a Nicolet IS 50 FT-IR spectrometer (Thermo Fisher Scientific, Waltham, MA, USA) operating in ATR mode.

XRD analysis of the analyzed samples was performed with CuKα1.2 nifilter using a Philips PW 1050 powder diffractometer (Philips Company, Groenewoudseweg 1, Amsterdam, The Netherlands) operating on the principle of Bragg–Brentano geometry, with a nifilter, using X-ray tubes with a copper anticathode, and using the radiation with a wavelength of λ_Cuα_ = 0.154185 nm.

The crystallinity X_c_ of the AgHA sample can be approximately determined using equation [33]:

X_c_ ≈ 1 − V_112/300_/I_300_, (1)

where V_112/300_ represents the intensity of the hollow between the diffraction peaks (112) and (300), and I_300_ is the intensity of the (300) diffraction peak.

### 2.3. Contact Angle Measurements

Contact angle measurements were performed with the Attension Theta tensiometer (Biolin Scientific AB, Västra Frölunda, Sweden), which consists of a light source, a camera, a liquid dispenser, and a sample stage. The CS-AgHA composite surfaces were placed on the sample stage.

A drop of distilled water (V ≈ 5 μL) was placed on the CS-AgHA composite surface, and the contact angle between the drop and the surface was measured. Eight measurements were taken for each material, from which the average contact angle value was calculated.

### 2.4. Roughness Measurements

We used profilometry to characterize the orthopedic surface topography. From imaging data, the quantitative evaluation of surface features was performed and from the statistical analysis, the roughness parameters of the orthopedic surfaces were determined. On each type of orthopedic surface, three-line measurements in the length of 5 mm were taken. The roughness data were analyzed and the arithmetic mean roughness (R_a_) and root mean square roughness (R_q_) were calculated.

We used the Form Talysurf Series 2 profilometer (Taylor-Hobson Ltd., Leicester, UK) with a resolution of 0.25 μm, 1 μm and 3 nm in the x, y and z directions. A set of parallel line scans was performed using a tip of 2 μm. The roughness data was processed with TalyMap Gold 6.2. A Gaussian cut-off filter of 0.8 mm was applied to separate the roughness from the waviness. The orthopedic surfaces were characterized in terms of height, space and hybrid parameters according to the ISO 25178 standard [34].

### 2.5. Bacterial Adhesion

#### 2.5.1. Bacteria

Following an 18 h incubation, the bacterial culture was used as the starting inoculum. The 18 h incubation period was selected based on a preliminary growth-curve experiment with *Staphylococcus aureus* ATCC 25923, in which the culture reached approximately 10^9^ CFU/mL at this time point [35]. Thus, a concentration of approximately 10^9^ CFU/mL was used as the expected starting bacterial density for the subsequent experiments.

#### 2.5.2. Crystal Violet Test to Determine the Adhesion of *S. aureus* to Various Coated Surfaces

A crystal violet (CV) test was performed to determine the adhesion of *S. aureus* on various coated surfaces. The sessile bacterial cells were determined by colorimetric measurement at 620 nm using a microplate reader (Tecan, Mannedorf/Zurich, Switzerland). The optical density measurement was proportional to the density of biofilms on the surfaces. The *S. aureus* reference strain ATCC 25923 was inoculated in 5 mL brain heart infusion (BHI) medium for overnight culture to obtain a suspension of 10^9^ cells/mL medium. Then an inoculum from bacteria by diluting the overnight culture (1:300) to an approximate cell concentration of 10^6^ CFU/mL was prepared. A sample of 5 mL of this suspension was incubated with four parallel samples of each sample material covered with CS, AgHA, CS:AgHA = 1:1, CS:AgHA = 1:2, CS:AgHA = 2:1, for 12 h. As a negative control, we added four samples to a six-well microtiter plate containing 5 mL of fresh growth medium BHI. After incubation, the samples were carefully rinsed three times with 5 mL of sterile phosphate buffered saline (PBS) and dried for 10 min at 600 C using a hair dryer. The adherent bacteria were stained with 10 μL 0.04% CV (Merck 115940) on the top of the samples. After 5 min of staining, the CV solution was removed and each sample was washed three times with PBS before drying with a hair dryer. The bound CV solution was then dissolved by adding 400 μL of 96% ethanol and 200 μL of this solution was transferred to a 96-well microplate to measure the absorbance.

With the quantitative method, the change in the number of viable cells was determined. *S. aureus* overnight culture was prepared by transferring one bacterial colony into 5 mL of brain heart infusion growth medium (BHI, Biolife, Milan, Italy) and incubated for 18 h at 37 °C to obtain 10^9^ CFU/mL. After incubation, the 10^5^ CFU/mL of *S. aureus* suspension was prepared in BHI. Pure titanium surfaces (1 cm × 1 cm) and surfaces treated with chitosan and chitosan with hydroxyapatite doped with Ag ions in different amounts were sterilized under UV light. The experiments were conducted in triplicates. The sterilized samples were placed into a 24-well plate and covered with 100 µL of 10^5^ CFU/mL *S. aureus* suspension and incubated for 24 h at 37 °C. The samples were, after incubation, washed twice with 1 mL of 0.9% NaCl solution and the washing solution was collected, and after a series of dilutions, was used for the determination of viable planktonic *S. aureus* cells. The washed samples were added with sterile forceps into 2.5 mL of 0.9% NaCl solution, placed into an ultrasonic water bath and sonicated for 5 min. Following sonication, the samples were vortexed at the highest speed for 15 s. The solution was collected and after a series of dilutions used for the determination of the viable adhered *S. aureus* cells. The number of viable cells was determined by spotting 10 µL of diluted bacterial suspensions onto plate count agar plates, which were incubated for 18 h at 37 °C. The total number of viable *S. aureus* cells was determined from the sum of planktonic and adhered cells. The reduction percentage (*P*) in the *S. aureus* viability was calculated:

$$P=(1\;-\;\frac{B}{A})\;\times 100$$

from the number of viable *S. aureus* cells on the blank control group—pure titanium surfaces (A) and the number of viable *S. aureus* cells on the surfaces treated with chitosan and chitosan with hydroxyapatite doped with Ag ions in different amounts (B).

Furthermore, the antibacterial activity of the pure hydroxyapatite sample—the control sample—and hydroxyapatite sample doped with Ag ions was determined. Sterile physiological saline solution (9.9 mL) was inoculated with 0.1 mL of overnight *S. aureus* bacterial culture. The 100 mg sample of hydroxyapatite doped with Ag was sterilized in an autoclave and added to a sterile tube with the above-described bacterial suspension. The suspensions were incubated at 37 °C with shaking. The number of viable cells was determined after 0, 1, 2, and 4 h. After a series of dilutions, 10 μL of the *S. aureus* suspension was loaded on plate count agar plates and incubated at 37 °C for 18 h to determine the number of viable *S. aureus* cells. The tests were repeated in triplicate. Pure titanium discs were used as the blank control group, chitosan-coated titanium as the comparative control, and chitosan–AgHA composites as the test coatings. In addition, AgHA and undoped HA powders were evaluated separately as positive and negative controls, respectively, to distinguish the antibacterial contribution of Ag ions from the chitosan matrix.

The detection limit of the colony-counting method was determined by the minimum detectable colony (1 CFU) on a 10 μL plated volume, corresponding to 1 × 10^2^ CFU/mL (2 log_10_ CFU/mL). Values below this limit were considered below the detection threshold.

#### 2.5.3. Assessment of Viability with Live/Dead Staining

After incubation and washing, samples were stained with the LIVE/DEAD BacLight Bacterial Viability Kit (Invitrogen, Thermo Fisher Scientific, Waltham, MA, USA) according to the manufacturer’s instructions. Live cells were stained with SYTO 9 green fluorescent dye, while dead cells appeared red after staining with propidium iodide. The double-stained samples were examined using the Nikon Eclipse Ni fluorescence microscope (Nikon; Tokyo, Japan).

Quantitative analysis of bacterial coverage of the surfaces was performed using a scanning electron microscope (SEM, Quanta 650, Thermo Fisher Scientific, Waltham, MA, USA). The samples were coated with gold (BAL-TEC SCD 005, BALTEC AG, Pfäffikon, Switzerland) and the thickness of the gold layer was ~5 nm. The vacuum during gold coating was 5 × 10^−2^. The working distance of 10 mm at an accelerating voltage of 10 kV and a vacuum of 2 × 10^−4^ was used for imaging with the secondary electron sensor. In the SEM micrographs, the adherent bacteria were manually circled and converted to binary format images. The ImageJ software package (version 1.50b, 2015, Wayne Rasband, National Institutes of Health, Bethesda, MD, USA) was used for further analysis [36].

#### 2.5.4. Antibacterial Activity Study with the Disc Diffusion Method

The agar diffusion test was employed to evaluate the antimicrobial activity of the different chitosan samples. Bacterial inoculum was prepared and adjusted to 0.5 McFarland and swabbed on Mueller–Hinton agar plates. Standard paper discs were impregnated with the different chitosan samples and placed onto the surface of the Mueller–Hinton agar. Additionally, a paper disc impregnated with 1% acetic acid was used as a negative control. A gentamicin paper disk was used as a performance control. The agar plates were incubated for 24 h at 37 °C. After incubation, the average diameter of the inhibition zone (in mm) was evaluated.

### 2.6. Genotoxicity Testing

The genotoxicity of the materials was evaluated with human peripheral blood lymphocytes as a model system using the cytokinesis block micronucleus test according to ISO 10993-3 [30].

#### 2.6.1. Cell Culture and Exposure Protocol

The blood sample was obtained from a healthy, non-smoking young volunteer in accordance with protocols approved by the Institutional Ethics Committee (approval No. 116-5-5/2023-000, approved on 5 July 2023) and in compliance with current regulations [37]. Aliquots of heparinized whole blood (0.5 mL) were placed in culture tubes containing 4.5 mL of RPMI-1640 medium supplemented with 15% calf serum and 2% phytohemagglutinin (Invitrogen-Gibco, Paisley, UK). Twenty-four hours after the start of the culture, the cells were exposed to the pure Ti plates and Ti plates coated with CS-AgHA (weight ratio 2:1), which had previously been sterilized under the UV lamp for 30 min. A sufficient number of blood cell cultures were prepared to examine the frequency of micronuclei (MN) and a cytokinesis block proliferation index (CBPI). The untreated cultures served as controls. Three independent experiments were performed. The data obtained were pooled, and the results were expressed as the mean and standard deviation (SD) of the mean.

#### 2.6.2. The Cytokinesis-Block Micronucleus (CBMN) Test

The cytokinesis block micronucleus test is a sensitive and standardized method for the simultaneous assessment of genotoxic and cytotoxic effects of tested materials in cultured human lymphocytes [38,39].

##### Micronuclei Analysis

For micronuclei preparation, the CBMN assay of Fenech [40] was used according to the Organization for Economic Cooperation and Development (OECD) guideline 487 [41]. Cytochalasin B (Sigma-Aldrich, St. Louis, MO, USA) at a final concentration of 4 µg/mL was added to each culture 44 h after incubation to inhibit cytokinesis. The lymphocyte cultures were incubated for a further 28 h. Cells were collected by centrifugation and treated with hypotonic solution (0.56% KCl + 0.90% NaCl, mixed in equal volumes) at 37 °C. The cell suspension was fixed in methanol/acetic acid (3:1), washed three times with fixative, and dropped onto clean slides. The slides were air dried and stained with alkaline Giemsa. For each sample, at least 2000 binucleated cells were scored, and micronuclei were evaluated using an Optech microscope (Munich, Germany) at 400× or 1000× magnification.

##### Estimation of the Cytokinesis-Block Proliferation Index

The cytostatic effect of the materials was measured by the proportion of mononucleated, binucleated, and multinucleated cells. The CBPI was calculated according to the method of Surrales et al. [42] as follows: CBPI = [MI + 2MII + 3(MIII +MIV)]/N, where MI-MIV represent the number of cells with one to four nuclei, respectively, and N is the number of cells scored. At least 500 cells per slide were counted for their determination. The relative frequencies of mononucleated, binucleated, trinucleated, and tetranucleated cells in the culture provide an accurate method to quantify the effects on cell proliferation and cytotoxic or cytostatic activity of the treatment. Cytotoxicity was measured in accordance with OECD 487 [41] using the CBPI, where

% cytostasis = 100 − 100[(CBPI_T_ − 1)/(CBPI_C_ − 1)], where CBPI_T_ is the CBPI of the test compound, and CBPI_C_ is the CBPI of the untreated control cells.

According to the test guideline, cytotoxicity is identified as a reduction in CBPI. If the maximum concentration is based on cytotoxicity, the highest concentration should aim to achieve a cytotoxicity of (55 ± 5)%, i.e., to show a reduction in CBPI of up to (45 ± 5)% compared to the concurrent negative control [41].

### 2.7. Statistics

The statistical analysis of the individual parameters was carried out using the Statistics software package, version 7, for Microsoft Windows. The statistical analysis was performed using a one-way analysis of variance (ANOVA) and Student’s *t*-test. The *p*-value < 0.05 was considered significant.

## 3. Results and Discussion

In this work, we have developed a novel antibacterial biocomposite intended for coating titanium bone implants. All components of the composite are biodegradable, biocompatible, and non-toxic. CS contributes a local bacteriostatic effect, supporting initial infection prevention at the implantation site. The AgHA component provides a sustained antimicrobial effect through the controlled release of silver ions, enhancing the long-term antibacterial performance of the coating [32]. Furthermore, both CS and AgHA exhibit osteoconductive properties, which are essential for promoting bone cell adhesion, proliferation, and successful integration of the implant with surrounding bone tissue [43,44].

### 3.1. Synthesis and Characterization of the Composites

**Figure 1:**
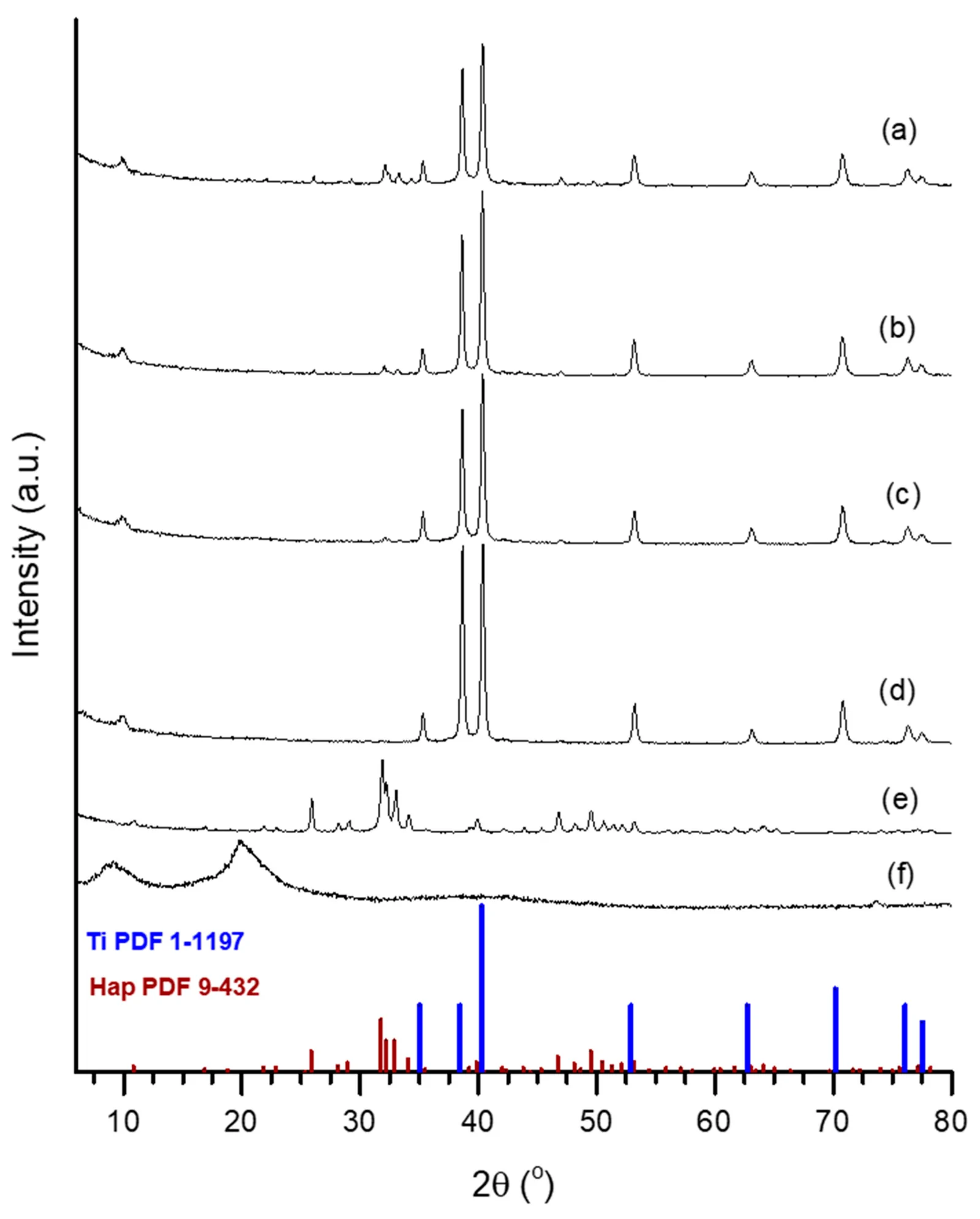
XRD diffractograms of composites CS:AgHA = 1:2 (**a**), CS:AgHA = 1:1 (**b**), CS:AgHA = 2:1 (**c**) on Ti plate. A thin film of CS on Ti plate (**d**). Pure AgHA (**e**) and pure CS (**f**). Ti PDF 1-1197 card corresponds to pure Ti (blue peaks) whereas Hap PDF 9-432 card corresponds to pure AgHA (red peaks).

XRD patterns of AgHA, CS and CS-AgHA composites are shown in Figure 1. The obtained AgHA powder crystallized as hydroxyapatite phase Ca_5_(PO_4_)_3_OH (PDF card no. 9-432) in the hexagonal space group P6_3/m_ (S.G. 176), as confirmed by XRD profiles in Figure 1e [32]. The crystallinity of the AgHA sample is 74%, according to Equation (1). Ti is identified on the card Ti PDF1-1197 as shown in Figure 1a–d. CS on Ti plate sample only shows titanium diffraction due to the small amount and weak intensity of CS peaks caused by its amorphous structure (Figure 1d) [45]. The presence of the pure AgHA phase sample can be seen by very weak peaks: (211) and (300) (Figure 1e). In the composite sample with the highest AgHA content (Figure 1a), the peaks originating from the apatite phase (211, 112, 300, and 202) are more pronounced.

**Figure 2:**
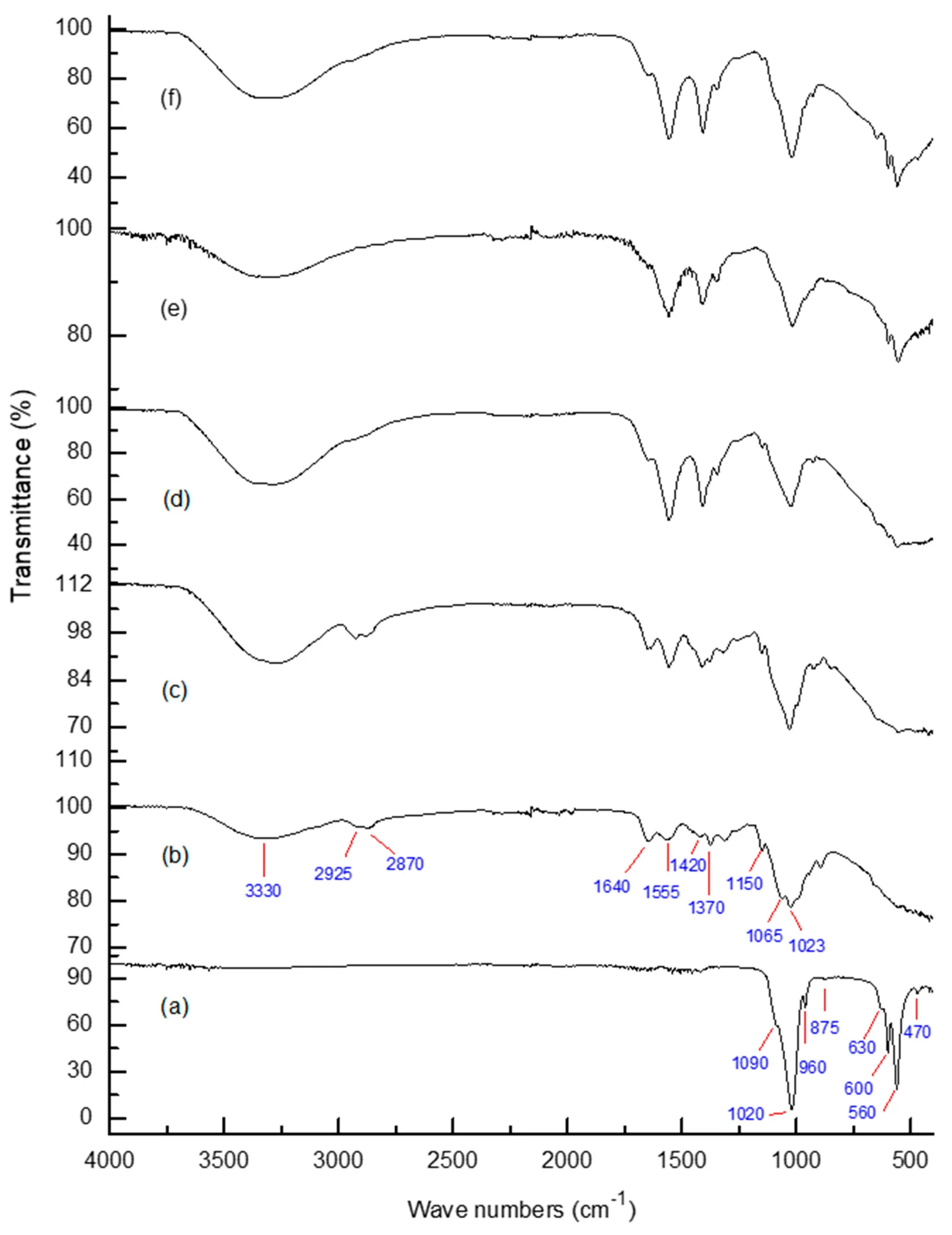
FTIR spectra of AgHA (**a**) and CS (**b**). Thin films on Ti plate: CS with glycerol (**c**), CS:AgHA = 2:1 (**d**), CS:AgHA = 1:1 (**e**), and CS:AgHA = 1:2 (**f**).

The FTIR spectra of the AgHA, CS-Ti, and CS-AgHA composites are illustrated in Figure 2. The AgHA sample contains characteristic bands for chemical groups of apatite compounds (Figure 2a). The phosphate groups are dominant in the spectrum. The strong band located at 1020 cm^−1^ and a shoulder at 1090 cm^−1^ resulted from ν3 P–O antisymmetric stretching vibrations. The band at 960 cm^−1^ reflects the P–O symmetric stretching (ν1). The 600–560 cm^−1^ doublet represents ν4 O–P–O bending vibrations. The weak band at about 470 cm^−1^ corresponds to the phosphate bending vibration. The small band at about 875 cm^−1^ (ν2) is attributed to (CO_3_)^2−^ ions and indicates the substitution of (PO_4_)^3−^ groups by (CO_3_)^2−^ groups and the formation of hydroxyapatite with carbonate (B-type) [32,46]. The carbonate content in the AgHA sample is low, as measured by the intensity of the absorption band. The shoulder at about 630 cm^−1^ originates from the stretching vibrations of the OH− groups that verify the presence of the hydroxyapatite phase. The spectrum of CS (Figure 2b) shows several characteristic bands [47]. The spectra display a broader peak at 3330 cm^−1^ arising due to the hydroxyl group and amino group stretching vibration overlap. The bands at 2925 cm^−1^ and 2870 cm^−1^ were due to C-H symmetric and asymmetric stretching, respectively. The band at about 1640 cm^−1^ belongs to amide I vibrations C=O stretching. The broad band at 1555 cm^−1^ represents the N-H (amide II band) of chitosan carbon chains. Bands at around 1420 cm^−1^ and 1370 cm^−1^ can be attributed to the CH_2_ bending and CH_3_ symmetrical deformations, respectively. The band at 1150 cm^−1^ is ascribed to asymmetric stretching of the C-O-C bridge. The bands at 1065 cm^−1^ and 1023 cm^−1^ are associated with C-O stretching.

The FTIR spectra of CS with glycerol on the Ti plate (Figure 2c) are similar to those of pure CS (Figure 2b). The bands are shifted due to hydrogen bonding and electrostatic interactions between CS and glycerol molecules. FTIR of the composite samples shows characteristic CS bands that are slightly shifted and altered due to interaction with glycerol molecules and with the surface of AgHA particles. The FTIR bands of the composite samples show characteristic bands of pure CS and the characteristic strong band at 1020 cm^−1^ and doublet present at 600–560 cm^−1^ of (PO_4_)^3-^ groups from the apatite phase (Figure 2d–f).

The contact angle measurements show that the surfaces are hydrophilic and lie very close to the boundary between hydrophilic and hydrophobic. The highest contact angle was measured for CS surfaces. The lowest contact angle was determined for the CS:AgHA = 2:1 ratio.

**Figure 3:**
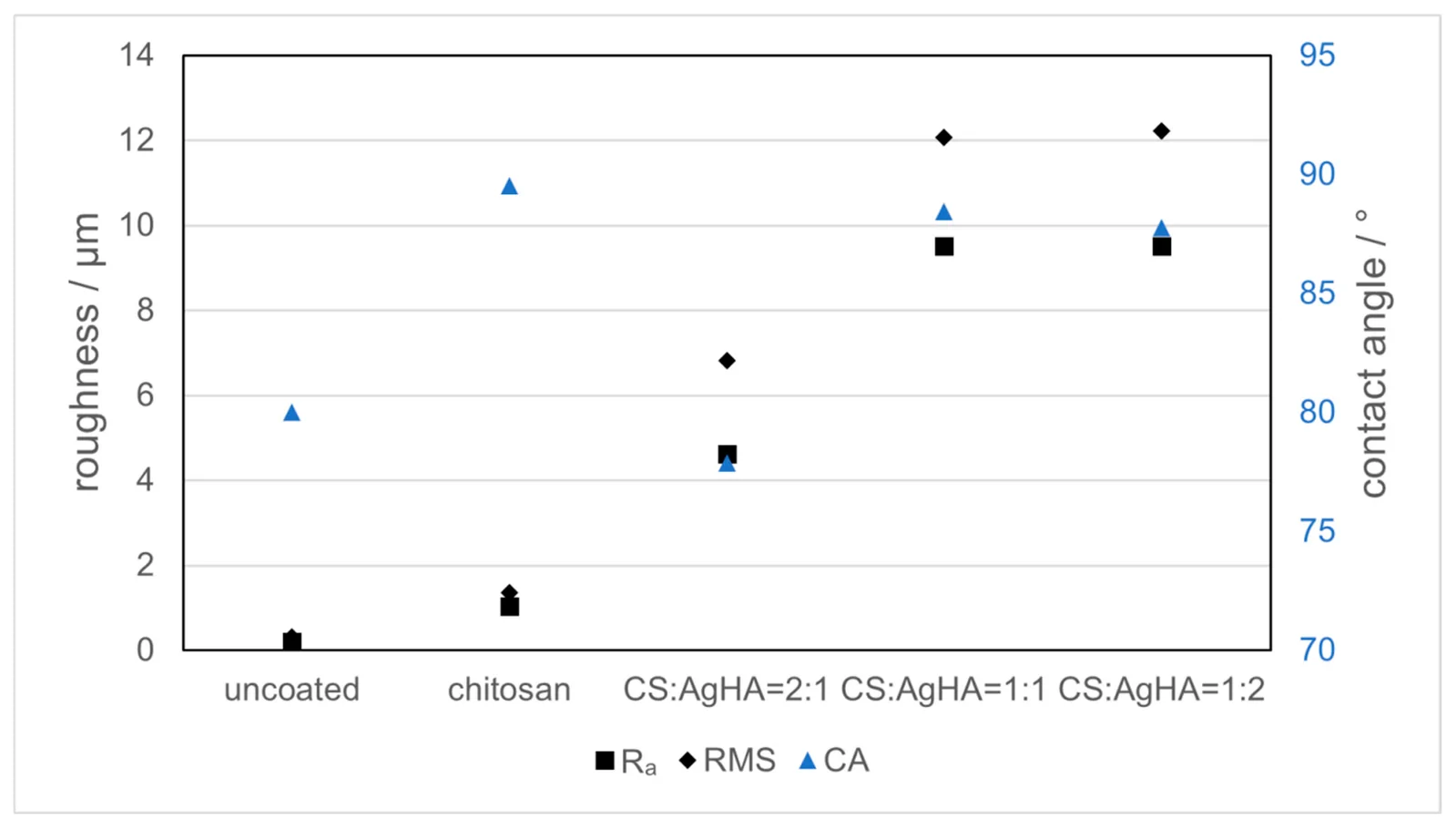
Surface roughness (µm) and contact angle θ (°) of uncoated, chitosan (CS), and CS-AgHA coated Ti surfaces with different CS:AgHA ratios.

The arithmetic mean roughness value of the CS surfaces is R_a_ = (1.04 ± 0.31) μm and the root mean square roughness value is R_q_ = (1.36 ± 0.42) μm. As the ratio of AgHA to CS increases, the surface roughness increases. The highest roughness was achieved for CS:AgHA = 1:1 and CS:AgHA = 1:2 (Figure 3). Namely the addition of AgHA nanoparticles makes the surface rougher due to the increasing number of nanoparticles.

Surface wettability is strongly influenced not only by the intrinsic surface chemistry but also by surface topography. According to the Wenzel model, when a liquid fully penetrates surface asperities, increasing roughness amplifies the intrinsic wetting behavior of the material, making hydrophilic surfaces more hydrophilic and hydrophobic surfaces more hydrophobic. In contrast, the Cassie–Baxter model describes a situation in which air pockets remain trapped beneath the liquid droplet, resulting in a composite solid–air interface and an apparent increase in contact angle. The present results suggest that the CS:AgHA coatings primarily follow a Wenzel-type wetting regime, as the incorporation of AgHA increased surface roughness while maintaining hydrophilic behavior. In particular, the CS:AgHA = 2:1 coating exhibited a substantially higher roughness (Ra = 4.63 ± 0.91 μm) than pure chitosan (Ra = 1.04 ± 0.31 μm), accompanied by a decrease in water contact angle from 89.54° to 77.88°, indicating enhanced wettability. This observation is consistent with the penetration of water into the surface microstructure and increased effective solid–liquid contact area. However, the further increase in AgHA content within the coating seems to negate this effect. Since bacterial adhesion is governed by a combination of physicochemical interactions, surface roughness and wettability should be considered together rather than independently. Rougher surfaces can provide additional shelter and attachment sites for microorganisms, whereas increased hydrophilicity may reduce the strength of bacterial adhesion by modifying interfacial free energy and hydration forces. Therefore, the observed antibacterial performance of the CS:AgHA coatings is likely governed by the combined effects of surface topography, wettability, and the antimicrobial action of released Ag^+^ ions. These findings support the concept that wettability and topography jointly govern bacterial adhesion and should be optimized simultaneously when designing antimicrobial implant coatings.

### 3.2. In Vitro Performance

To investigate the potential of Ti implants coated with CS and CS-AgHA as permanent implants, we determined their antibacterial performance and genotoxicity/cytotoxicity in vitro.

#### 3.2.1. Antibacterial Activity

Initial bacterial adhesion and subsequent colonization on the surface of a bone implant are crucial steps in the development of implant infection, which can lead to implant removal. Bacteria show an affinity to adhere to the surface of hydroxyapatite implants [48]. Several studies have shown that AgHA coatings exhibit antibacterial activities and do not show significant cytotoxic activity [28,49,50]. In vivo studies have shown that AgHA fillers for bone defects allow adequate cell proliferation and favorable bone repair without significant inflammatory responses [43,51]. CS, a biodegradable natural polymer, is particularly attractive as a component of bone implants, as it supports the attachment and proliferation of osteoblast cells and exhibits antimicrobial activity [52]. The molecular weight (MW) and degree of acetylation (DA) of CS are important factors that determine the rate and extent of biodegradation. When MW decreases and DA increases, biodegradation accelerates [53]. In this study, medium molecular weight was used, which is suitable for the production of bone implants [54].

**Figure 4:**
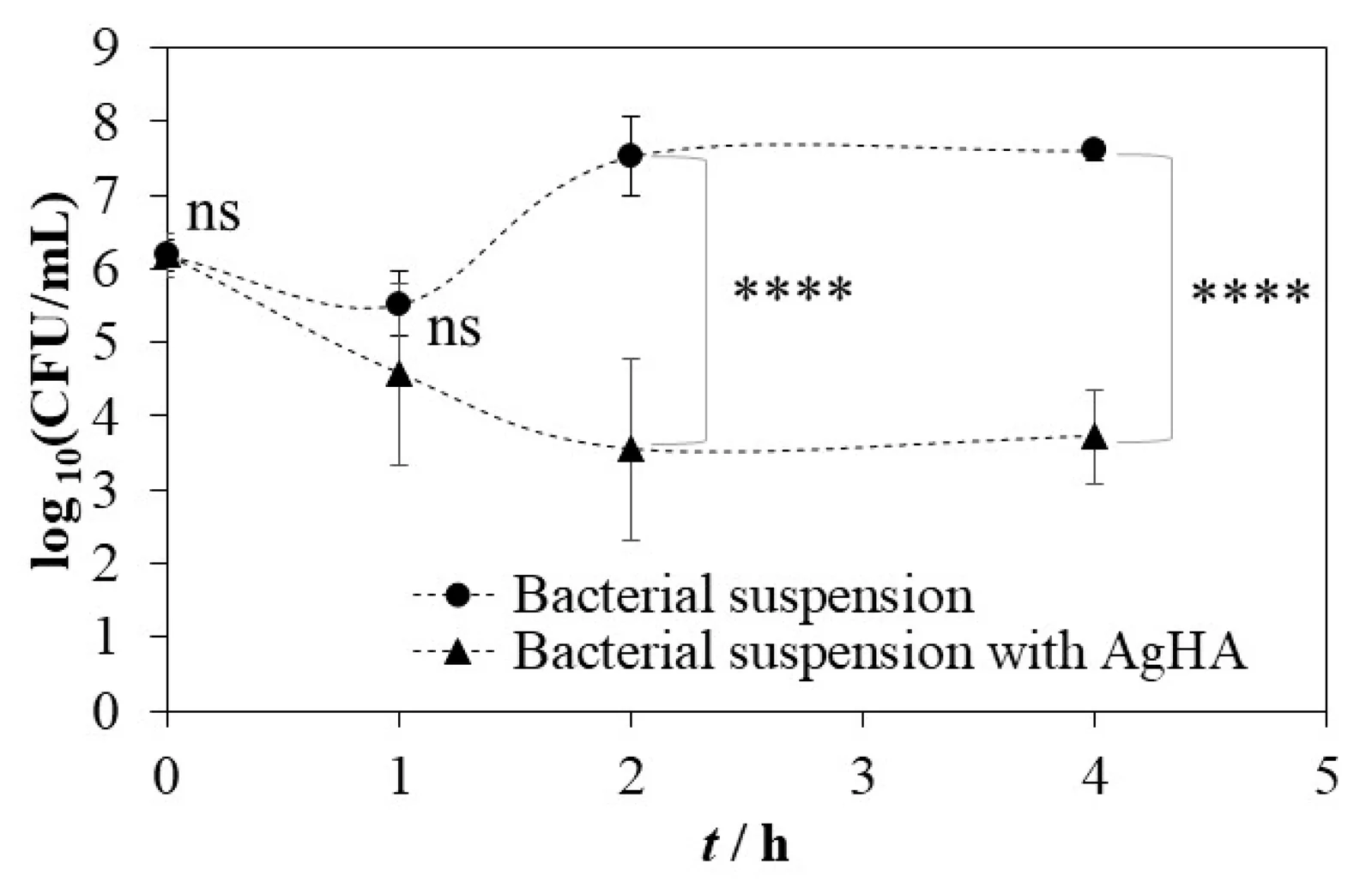
The number of viable *S.aureus* cells expressed as log_10_(CFU/mL) from the bacterial suspensions incubated with and without AgHA. Triplicate samples were used in the experiments. Significance was determined using a one-way ANOVA Tukey test of the logarithmic reduction in data points, with ns—not significant and **** *p* < 0.0001. The limit of detection (LOD) of the plate counting method was 1 × 10^2^ CFU/mL (2 log_10_ CFU/mL). Counts below this value were considered below detection limit.

The antibacterial activity of the AgHA on the viability of *S. aureus* in solution was determined by monitoring the change in the number of viable cells over 4 h (Figure 4). The results demonstrated that the number of viable cells in the suspension without the AgHA increased during the four hours of incubation from 1 × 10^6^ CFU/mL to 5 × 10^7^ CFU/mL. In the suspension with the added AgHA, the viability of the *S. aureus* cells decreased by (74.3 ± 6.2)% after one hour. In addition, the viability of the *S. aureus* cells had decreased by more than 99% after two hours and the number of cells did not increase after a further two hours of incubation (Figure 4).

**Figure 5:**
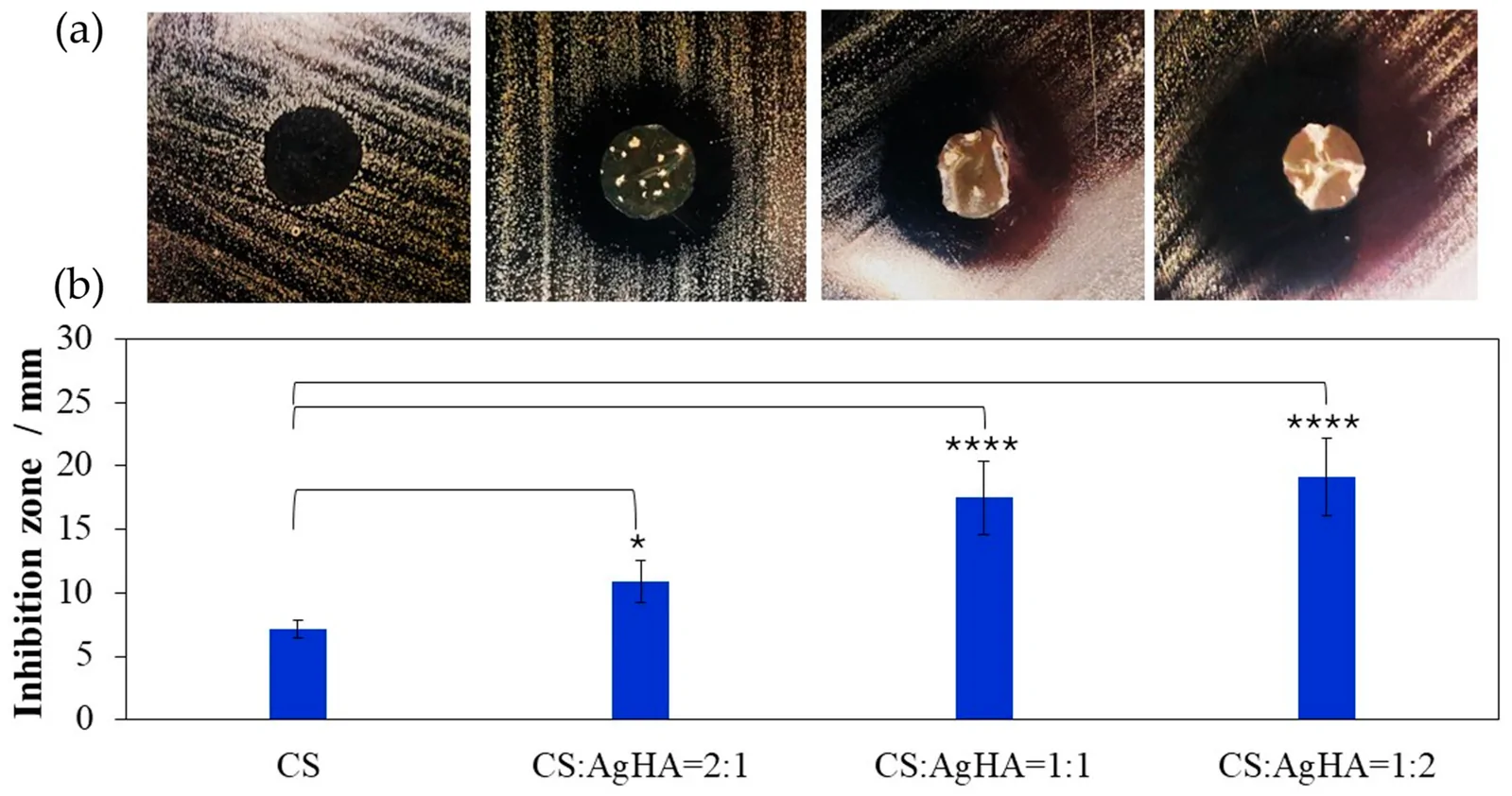
(**a**) Inhibition zones from the diffusion test with films made from CS and (**b**) CS-AgHA for different ratios CS:AgHA. Triplicate samples were used in the experiments. Significance was determined using a one-way ANOVA Tukey test of the inhibition zone compared to pure chitosan with * *p* ≤ 0.0229, and **** *p* < 0.0001.

The leaching effect of Ag ions from the CS and HA discs on the development of an inhibition zone was carried out using the disc diffusion test (Figure 5). The results showed that no zone of inhibition developed in the case of pure CS (Figure 5a). However, the bacteria did not grow under the discs, indicating an antibacterial effect due to direct contact with the bacteria. On the other hand, all CS-AgHA exhibited inhibition zones caused by the leaching of Ag ions into the agar (Figure 5a). The inhibition zone increased with the increased amount of AgHA (Figure 5b). The most effective activity was observed for the sample CS:AgHA = 1:2 with a diameter of the inhibition zone of (19.1 ± 3.0) mm.

**Figure 6:**
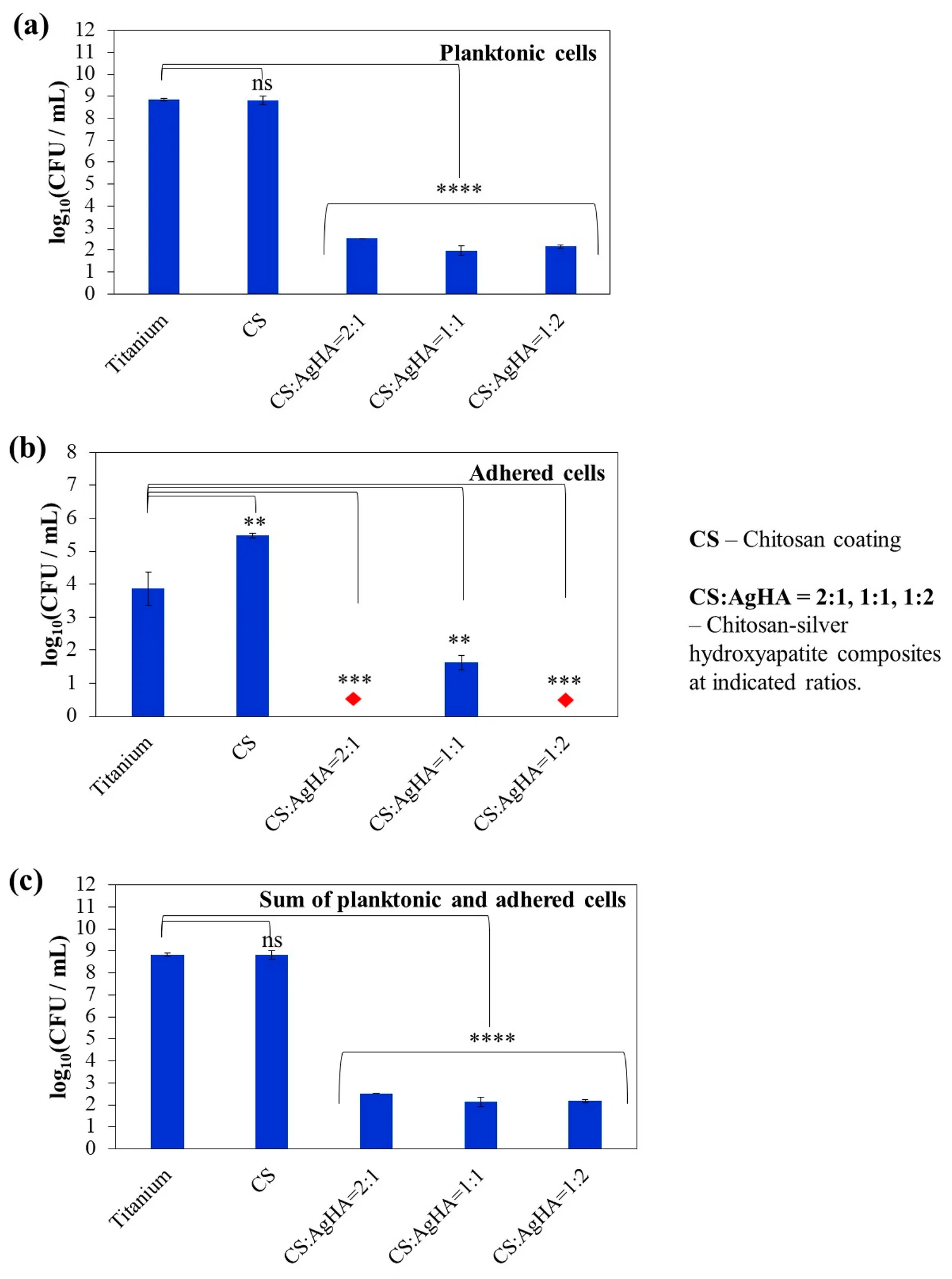
Average logarithmic bacterial count per milliliter (CFU/mL) on Ti surfaces coated with CS—chitosan and CS-AgHA with different ratios CS:AgHA against *S. aureus*. Shown are the number of planktonic (**a**), adherent (**b**), and total (**c**) *S. aureus* cells. Each bar represents the mean ± SD of three replicates. Groups are indicated on the x-axis as follows: Ti—uncoated titanium; CS—chitosan coating; CS:AgHA = 2:1, 1:1, 1:2—chitosan–silver hydroxyapatite composites at the indicated weight ratios. Triplicate samples were used in the experiments. Significance was determined using a one-way ANOVA Tukey test of the logarithmic reduction in data points compared to uncoated Ti surfaces with ** *p* ≤ 0.0020, *** *p* ≤ 0.0005 and **** *p* < 0.0001.

The number of viable *S. aureus* cells was determined in different amounts on the pure Ti surfaces, the surfaces treated with CS and the CS-AgHA surfaces (Figure 6). A one-way analysis of variance (ANOVA) was performed to statistically determine the significance of the difference between uncoated and coated Ti surfaces. The number of viable planktonic *S. aureus* cells (Figure 6a, Table 1) was significantly lowered (>99%) after the application of CS and CS-AgHA with different ratios of CS:AgHA (*p* < 0.0001). Furthermore, the number of adherent viable cells was also significantly lowered (>99%) compared to the pure Ti surfaces in the case of CS (*p* = 0.0002) and CS-AgHA in a ratio of 2:1 (*p* < 0.0001), 1:1 (*p* = 0.0020) and 1:2 (*p* = 0.0005). To compare the overall effect, the viability of the sum of *S. aureus* cells (sum of planktonic and adherent cells) was determined (Figure 6c, Table 1). The results showed that, compared to the Ti surfaces, the viability of *S. aureus* cells decreased significantly (>99.9%) after surface coating with CS and CS-AgHA for different CS:AgHA ratios (*p* < 0.0001). No statistically significant difference was observed between the CS and CS-AgHA surfaces with different CS:AgHA ratios (*p* > 0.4758).

While the present study demonstrates significant antibacterial performance of CS-AgHA coatings up to 24 h of bacterial exposure, future investigations will focus on extended incubation periods (≥7 days) to evaluate the long-term durability and sustained antibacterial activity of the coatings under simulated physiological conditions.

**Figure 7:**
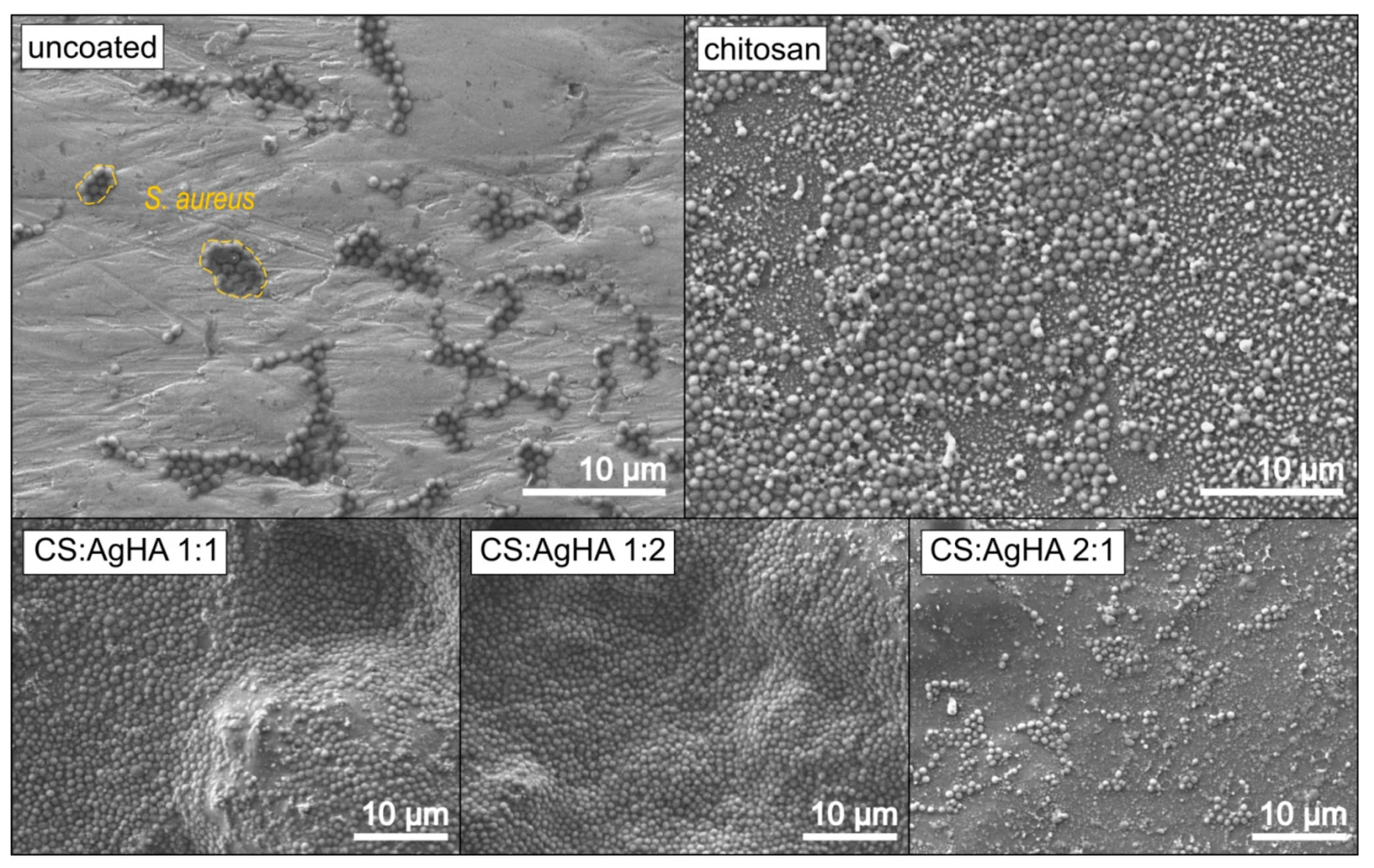
SEM micrographs of adherent bacteria *S. aureus* to different coatings.

SEM micrographs of adherent bacteria *S. aureus* to different coatings are presented in Figure 7. While uncoated surfaces are not densely colonized and chitosan-coated surfaces are densely colonized by viable cocci, the AgHA-containing coatings exhibit disrupted bacterial clusters and deformed cell morphologies, suggesting membrane damage and impaired biofilm formation. The observed antibacterial performance of CS-AgHA coatings can be attributed to the synergistic effects of chitosan and Ag^+^ ions. The pronounced reduction (>99%) in *S. aureus* viability observed for AgHA-containing samples can be attributed to the bactericidal action of Ag^+^ ions released from the HA. Previously, the mechanism of Ag^+^ action was researched; it showed that released ions first bind to the negatively charged components of the Gram-positive cell (teichoic acids, peptidoglycans) and sulfhydryl-containing membrane proteins, thus increasing the membrane permeability, surface damage and cell aggregation visible in Figure 7 [55]. Subsequently, Ag^+^ ions penetrate into the cytoplasm and inactivate respiratory chain dehydrogenases and cytochromes, ATP depletion, interfere with DNA replication through Ag^+^ binding to phosphate and nitrogen donor sites, resulting in the time-kill experiment in suspension (Figure 4) [56]. Antibacterial properties of AgHA overdominate the roughness effect. Comparable mechanisms have been reported for silver-containing HA and Ag-based antibacterial coatings [11,57]. The increase in the inhibition zone with increasing AgHA fraction further confirms that the diffusion of Ag^+^ ions is responsible for the bactericidal effect. Chitosan’s positively charged amino groups hinder bacterial adhesion via electrostatic repulsion, while the gradual release of Ag^+^ ions from the hydroxyapatite matrix exerts a direct bactericidal effect on both planktonic and surface-associated cells. The enlarged inhibition zones and the marked decrease in viable CFU counts support the presence of ion-mediated killing. Although detailed Ag^+^ release kinetics and bacterial membrane integrity assays were not conducted in this study, future work will focus on quantifying ion release and visualizing bacterial membrane disruption and biofilm structure to further substantiate the antibacterial mechanism. In the present work, antibacterial performance was evaluated against *Staphylococcus aureus*, a representative Gram-positive pathogen and a major cause of implant-associated infections. While these results clearly demonstrate strong antibacterial efficacy, future studies will include additional clinically relevant bacterial strains, such as *Staphylococcus epidermidis*, *Pseudomonas aeruginosa*, and *MRSA*, to validate the broad-spectrum antibacterial potential of CS-AgHA coatings.

#### 3.2.2. Genotoxicity/Cytotoxicity Testing

The potential of synthetic materials for the development of orthopedic implants requires an investigation of their genotoxicity. Genotoxicity has been assessed using the cytokinesis-block micronucleus test that enables evaluation of the presence and the extent of the test substance-induced DNA damage, cytostasis, and cytotoxicity [58]. The CBMN assay has become one of the standard cytogenetic tests for genotoxicity testing in human and mammalian cells, as recommended in ISO 10993-3 [30], enabling simultaneous detection of genotoxicity and cytotoxicity.

In this study, Ti plates coated with CS:AgHA = 2:1 were selected for genotoxicity and cytotoxicity evaluation based on their favorable surface and antibacterial characteristics. In particular, the CS:AgHA = 2:1 coating exhibited the lowest surface roughness among the CS-AgHA coatings, while maintaining the strong antibacterial activity observed in the other CS-AgHA composites. It has been reported that surface roughness significantly influences cellular behavior, with lower roughness shown to enhance cell proliferation and biocompatibility in biomedical implants [59]. Plates made of pure Ti were also analyzed for comparison. The study was performed using human peripheral blood lymphocytes as a model system, according to OECD 487 standards [41]. Representative micrographs showing mononucleated, binucleated, and polynucleated cells, as well as a binucleated cell with a micronucleus after Giemsa staining, are presented in Figure S1.

As shown in Table 2 and Figure S2, both the pure Ti plates and the Ti plates coated with CS:AgHA = 2:1 did not lead to any significant changes in the incidence of MN compared to the untreated control. No significant differences were found between pure Ti plates and Ti plates coated with CS:AgHA = 2:1. Analysis of the CBPI values showed that the Ti plates significantly reduced the cell proliferation potential compared to the untreated control (*p* < 0.001). The effect of Ti plates coated with CS:AgHA = 2:1 on cell proliferation was similar (*p* < 0.01). No significant differences were observed between pure Ti plates and Ti plates coated with CS:AgHA = 2:1.

Further analysis of the cytotoxic/cytostatic effects of the tested materials showed that treatment of lymphocytes with pure Ti plates and Ti plates coated with CS:AgHA = 2:1 induced cell-growth inhibition (the percentage of cytostasis was 14.17 ± 0.68% and 15.35 ± 0.68%, respectively). These results indicate cell viability above 84% compared with the untreated control. No significant differences in the percentage of cytostasis were observed between the pure Ti plates and Ti plates coated with CS:AgHA = 2:1. According to ISO 10993 [30], a cell viability of 70% is considered the lower limit for cytocompatibility of the samples.

Based on these findings, neither pure Ti nor Ti coated with CS:AgHA at a 2:1 weight ratio showed an apparent potential to induce genotoxic effects under the experimental conditions applied. The observed cytostatic effects were limited, with cell viability remaining at approximately 84%. These findings provide evidence of favorable biocompatibility of the tested CS:AgHA = 2:1 coating in the human peripheral blood lymphocyte model.

CS and HA are recognized as biocompatible materials and are widely used in biomedical applications. However, the biocompatibility of silver-coated biomaterials may depend on silver concentration, its distribution within or on the coating surface, and the rate of silver ion release into surrounding biological fluids [60]. In our study, coating Ti plates with CS:AgHA = 2:1 maintained favorable cytocompatibility while providing appropriate antibacterial protection. Similar results have been reported in studies investigating CS-AgNPs and AgHANPs composite coatings [56,61].

A key limitation of our genotoxicity/cytotoxicity assessment is its reliance on human peripheral blood lymphocytes from a single healthy donor, evaluated exclusively for the CS:AgHA = 2:1 formulation. Although these results offer valuable preliminary insights into the cytogenetic response, further studies incorporating multiple donors and bone-relevant cell models are required to comprehensively characterize the biological safety of these materials. Ultimately, balancing strong antibacterial efficacy with robust biocompatibility remains a promising strategy for advancing orthopedic implant coatings.

## 4. Conclusions

The design of orthopedic implants and their functional properties are very important factors that enable their clinical application. With regard to antibacterial properties and biocompatibility, mixtures of chitosan and silver-doped hydroxyapatite coatings on Ti surfaces were prepared. The results show that the new coatings can reduce the bacterial adhesion of *S. aureus*. The CS:AgHA = 2:1 coating showed a favorable profile, combining antibacterial activity with high cell viability (>84%) and no genotoxic effects. These findings support its further investigation as an antibacterial, biocompatible coating for orthopedic Ti implants.

## Figures and Tables

**Table 1:** Percentage reduction (*P*) in viability of planktonic, adherent, and total *S. aureus* cells on chitosan CS and CS-AgHA for different ratios CS:AgHA compared to uncoated Ti surfaces.

	*P* (Planktonic) %	*P* (Adhered)/%	*P* (Sum)/%
CS	100 ± 0	99.98 ± 0.03	99.98 ± 0.03
CS:AgHA = 2:1	100 ± 0	100 ± 0	99.99 ± 0.01
CS:AgHA = 1:1	100 ± 0	99.94 ± 0.02	99.94 ± 0.02
CS:AgHA = 1:2	99.99 ± 0.01	99.99 ± 0	99.98 ± 0.01

**Table 2:** The incidence of MN, CBPI, and the cell inhibition rate (%) in cultured human lymphocytes exposed to Ti and Ti coated with CS:AgHA = 2:1.

Group	MN Incidence Rate	MN Incidence Rate Mean ± S.D.	CBPI	CBPI Mean ± S.D.	Cell Inhibition Rate (%)	Cell Inhibition Rate (%) Mean ± S.D.
Untreated Control	2.83	2.85 ± 0.25	1.85	1.85 ± 0.01	/	/
	3.11		1.84			
	2.61		1.85			
Pure Ti	3.25	3.16 ± 0.11	1.73	1.73 ± 0.01 *	13.78	14.17 ± 0.68
	3.18		1.72		14.96	
	3.04		1.73		13.78	
CS:AgHA = 2:1	3.01	3.05 ± 0.10	1.72	1.72 ± 0.01 *	14.96	15.35 ± 0.68
	2.98		1.72		14.96	
	3.16		1.71		16.14	

Cell inhibition rate = cytostasis (%), calculated from CBPI values (OECD TG 487). Data from three independent experiments were pooled, and values are expressed as the mean ± standard deviation (SD). * *p* < 0.05 compared to the untreated control; no label, *p* > 0.05.

## Data Availability

The original contributions presented in this study are included in the article/Supplementary Material. Further inquiries can be directed to the corresponding author.

## Supplementary Materials

The following supporting information can be downloaded at: https://www.mdpi.com/article/10.3390/molecules31193537/s1, Figure S1: Micrographs showing mononucleated, binucleated and polynucleated cells (a), normal binucleated cell (b), and binucleated cell with micronucleus (c) following Giemsa staining; Figure S2: Incidence of micronuclei (mean±SD) and cytokinesis-block proliferation index (mean±SD) in lymphocytes exposed to the Ti and Ti/CS/AgHA. The values are expressed as a percentage of control, set to 100%.

## References

[1] National Healthcare Safety Network, Centers for Disease Control and Prevention Surgical Site Infection [SSI] Event. http://www.cdc.gov/nhsn/pdfs/pscmanual/9pscssicurrent.pdf.

[2] Anderson D.J., Podgorny K., Berríos-Torres S.I., Bratzler D.W., Dellinger E.P., Greene L., Nyquist A.-C., Saiman L., Yokoe D.S., Maragakis L.L. (2014). Strategies to prevent surgical site infections in acute care hospitals: 2014 update. Infect. Control Hosp. Epidemiol..

[3] Bratzler D.W., Dellinger E.P., Olsen K.M., Perl T.M., Auwaerter P.G., Bolon M.K., Fish D.N., Napolitano L.M., Sawyer R.G., Slain D. (2013). Clinical practice guidelines for antimicrobial prophylaxis in surgery. Am. J. Health-Syst. Pharm..

[4] Trampuz A., Widmer A.F. (2006). Infections associated with orthopedic implants. Curr. Opin. Infect. Dis..

[5] Filipović U., Dahmane R.G., Ghannouchi S., Zore A., Bohinc K. (2020). Bacterial adhesion on orthopedic implants. Adv. Colloid Interface Sci..

[6] Shekhawat D., Singh A., Banerjee M.K., Singh T., Patnaik A. (2021). Bioceramic composites for orthopaedic applications: A comprehensive review of mechanical, biological, and microstructural properties. Ceram. Int..

[7] Piras A.M., Esin S., Benedetti A., Maisetta G., Fabiano A., Zambito Y., Batoni G. (2019). Antibacterial, Antibiofilm, and Antiadhesive Properties of Different Quaternized Chitosan Derivatives. Int. J. Mol. Sci..

[8] Chua P.-H., Neoh K.-G., Kang E.-T., Wang W. (2008). Surface functionalization of titanium with hyaluronic acid/chitosan polyelectrolyte multilayers and RGD for promoting osteoblast functions and inhibiting bacterial adhesion. Biomaterials.

[9] Li B., Xia X., Guo M., Jiang Y., Li Y., Zhang Z., Liu S., Li H., Liang C., Wang H. (2019). Biological and antibacterial properties of the micro-nanostructured hydroxyapatite/chitosan coating on titanium. Sci. Rep..

[10] Hou W., Shao L., Lou N., Shen Z., Piao Z., Ding C., Zhou Z. (2026). Improvements in surface integrity, corrosion and wear resistances of TA2 titanium alloy via cool-assisted ball burnishing. Tribol. Int..

[11] Cheng Y.F., Zhang J.Y., Wang Y.B., Li C.M., Lu Z.S., Hu X.F., Xu L.Q. (2019). Deposition of catechol-functionalized chitosan and silver nanoparticles on biomedical titanium surfaces for antibacterial application. Mater. Sci. Eng. C.

[12] Jin S., Li J., Wang J., Jiang J., Zuo Y., Li Y., Yang F. (2018). Electrospun silver ion-loaded calcium phosphate/chitosan antibacterial composite fibrous membranes for guided bone regeneration. Int. J. Nanomed..

[13] Liu P., Hao Y., Zhao Y., Yuan Z., Ding Y., Cai K. (2017). Surface modification of titanium substrates for enhanced osteogenetic and antibacterial properties. Colloids Surf. B Biointerfaces.

[14] Swartjes J.J.T.M., Sharma P.K., van Kooten T.G., van der Mei H.C., Mahmoudi M., Busscher H.J., Rochford E.T.J. (2015). Current Developments in Antimicrobial Surface Coatings for Biomedical Applications. Curr. Med. Chem..

[15] Yuan Z., Huang S., Lan S., Xiong H., Tao B., Ding Y., Liu Y., Liu P., Cai K. (2018). Surface engineering of titanium implants with enzyme-triggered antibacterial properties and enhanced osseointegration in vivo. J. Mater. Chem. B.

[16] Yuan Z., Liu P., Hao Y., Ding Y., Cai K. (2018). Construction of Ag-incorporated coating on Ti substrates for inhibited bacterial growth and enhanced osteoblast response. Colloids Surf. B Biointerfaces.

[17] Confederat L.G., Tuchilus C.G., Dragan M., Sha’at M., Dragostin O.M. (2021). Preparation and Antimicrobial Activity of Chitosan and Its Derivatives: A Concise Review. Molecules.

[18] Lin W.-T., Zhang Y.-Y., Tan H.-L., Ao H.-Y., Duan Z.-L., He G., Tang T.-T. (2016). Inhibited Bacterial Adhesion and Biofilm Formation on Quaternized Chitosan-Loaded Titania Nanotubes with Various Diameters. Materials.

[19] Richert L., Lavalle P., Payan E., Shu X.Z., Prestwich G.D., Stoltz J.-F., Schaaf P., Voegel J.-C., Picart C. (2004). Layer by Layer Buildup of Polysaccharide Films: Physical Chemistry and Cellular Adhesion Aspects. Langmuir.

[20] Zhang X., Zhang G., Zhang H., Li J., Yao X., Tang B. (2018). Surface immobilization of heparin and chitosan on titanium to improve hemocompatibility and antibacterial activities. Colloids Surf. B Biointerfaces.

[21] Carlson R.P., Taffs R., Davison W.M., Stewart P.S. (2008). Anti-biofilm properties of chitosan-coated surfaces. J. Biomater. Sci. Polym. Ed..

[22] Costa E.M., Silva S., Vicente S., Veiga M., Tavaria F., Pintado M.M. (2017). Chitosan as an effective inhibitor of multidrug resistant *Acinetobacter baumannii*. Carbohydr. Polym..

[23] Huang J., Cheng Y., Wu Y., Shi X., Du Y., Deng H. (2019). Chitosan/tannic acid bilayers layer-by-layer deposited cellulose nanofibrous mats for antibacterial application. Int. J. Biol. Macromol..

[24] Raphel J., Holodniy M., Goodman S.B., Heilshorn S.C. (2016). Multifunctional coatings to simultaneously promote osseointegration and prevent infection of orthopaedic implants. Biomaterials.

[25] Romanò C.L., Scarponi S., Gallazzi E., Romanò D., Drago L. (2015). Antibacterial coating of implants in orthopaedics and trauma: A classification proposal in an evolving panorama. J. Orthop. Surg. Res..

[26] Sánchez-Gálvez J., Martínez-Isasi S., Gómez-Salgado J., Rumbo-Prieto J.M., Sobrido-Prieto M., Sánchez-Hernández M., García-Martínez M., Fernández-García D. (2024). Cytotoxicity and concentration of silver ions released from dressings in the treatment of infected wounds: A systematic review. Front. Public Health.

[27] Honda M., Kawanobe Y., Ishii K., Konishi T., Mizumoto M., Kanzawa N., Matsumoto M., Aizawa M. (2013). In vitro and in vivo antimicrobial properties of silver-containing hydroxyapatite prepared via ultrasonic spray pyrolysis route. Mater. Sci. Eng. C.

[28] Ueno M., Miyamoto H., Tsukamoto M., Eto S., Noda I., Shobuike T., Kobatake T., Sonohata M., Mawatari M. (2016). Silver-Containing Hydroxyapatite Coating Reduces Biofilm Formation by Methicillin-Resistant *Staphylococcus aureus* In Vitro and In Vivo. BioMed Res. Int..

[29] Ribeiro M., Monteiro F.J., Ferraz M.P. (2012). Infection of orthopedic implants with emphasis on bacterial adhesion process and techniques used in studying bacterial-material interactions. Biomatter.

[30] (2014). Biological Evaluation of Medical Devices—Part 3: Tests for Genotoxicity, Carcinogenicity and Reproductive Toxicity.

[31] Huzum B., Puha B., Necoara R.M., Gheorghevici S., Puha G., Filip A., Sirbu P.D., Alexa O. (2021). Biocompatibility assessment of biomaterials used in orthopedic devices: An overview (Review). Exp. Ther. Med..

[32] Stanić V., Janaćković D., Dimitrijević S., Tanasković S.B., Mitrić M., Pavlović M.S., Krstić A., Jovanović D., Raičević S. (2011). Synthesis of antimicrobial monophase silver-doped hydroxyapatite nanopowders for bone tissue engineering. Appl. Surf. Sci..

[33] Landi E., Tampieri A., Celotti G., Sprio S. (2000). Densification behaviour and mechanisms of synthetic hydroxyapatites. J. Eur. Ceram. Soc..

[34] (2021). Geometrical product specifications (GPS) — Surface texture: Areal — Part 2: Terms, definitions and surface texture parameters.

[35] Bohinc K., Dražić G., Fink R., Oder M., Jevšnik M., Nipič D., Godič-Torkar K., Raspor P. (2014). Available surface dictates microbial adhesion capacity. Int. J. Adhes. Adhes..

[36] Schneider C.A., Rasband W.S., Eliceiri K.W. (2012). NIH Image to ImageJ: 25 years of image analysis. Nat. Methods.

[37] Law on Health Care, Official Gazette of the Republic of Serbia, No. 25/2019-40. https://pravno-informacioni-sistem.rs/eli/rep/sgrs/skupstina/zakon/2019/25/2/reg.

[38] Bolognesi C., Fenech M. (2019). Micronucleus Cytome Assays in Human Lymphocytes and Buccal Cells. Genotoxicity Assessment.

[39] Ruiz-Ruiz B., Arellano-García M.E., Radilla-Chávez P., Salas-Vargas D.S., Toledano-Magaña Y., Casillas-Figueroa F., Luna Vazquez-Gomez R., Pestryakov A., García-Ramos J.C., Bogdanchikova N. (2020). Cytokinesis-Block Micronucleus Assay Using Human Lymphocytes as a Sensitive Tool for Cytotoxicity/Genotoxicity Evaluation of AgNPs. ACS Omega.

[40] Fenech M. (1993). The cytokinesis-block micronucleus technique: A detailed description of the method and its application to genotoxicity studies in human populations. Mutat. Res.-Fundam. Mol. Mech. Mutagen..

[41] OECD (2023). Test No. 487: In Vitro Mammalian Cell Micronucleus Test. OECD Guidelines for the Testing of Chemicals, Section 4.

[42] Surrallés J., Xamena N., Creus A., Catalán J., Norppa H., Marcos R. (1995). Induction of micronuclei by five pyrethroid insecticides in whole-blood and isolated human lymphocyte cultures. Mutat. Res./Genet. Toxicol..

[43] Tian B., Chen W., Yu D., Lei Y., Ke Q., Guo Y., Zhu Z. (2016). Fabrication of silver nanoparticle-doped hydroxyapatite coatings with oriented block arrays for enhancing bactericidal effect and osteoinductivity. J. Mech. Behav. Biomed. Mater..

[44] Tian Y., Wu D., Wu D., Cui Y., Ren G., Wang Y., Wang J., Peng C. (2022). Chitosan-Based Biomaterial Scaffolds for the Repair of Infected Bone Defects. Front. Bioeng. Biotechnol..

[45] Tripathy P.K., Patra A.K., Sastry P.U. (2008). Studies on pore morphology of titanium and its oxide by small angle neutron scattering. Pramana.

[46] Golubevas R., Stankeviciute Z., Zarkov A., Golubevas R., Hansson L., Raudonis R., Kareiva A., Garskaite E. (2020). Acrylate–gelatin–carbonated hydroxyapatite (cHAP) composites for dental bone-tissue applications. Mater. Adv..

[47] Anicuta S.G., Dobre L., Stroescu M., Jipa I.M. (2010). Fourier transform infrared (FTIR) spectroscopy for characterization of antimicrobial films containing chitosan. Analele Univ. Oradea Fasc. Ecotoxicol. Zooteh. Tehnol. Ind. Aliment..

[48] An Y.H., Friedman R.J. (1998). Concise review of mechanisms of bacterial adhesion to biomaterial surfaces. J. Biomed. Mater. Res..

[49] Guimond-Lischer S., Ren Q., Braissant O., Gruner P., Wampfler B., Maniura-Weber K. (2016). Vacuum plasma sprayed coatings using ionic silver doped hydroxyapatite powder to prevent bacterial infection of bone implants. Biointerphases.

[50] Iconaru S.L., Predoi D., Ciobanu C.S., Motelica-Heino M., Guegan R., Bleotu C. (2022). Development of Silver Doped Hydroxyapatite Thin Films for Biomedical Applications. Coatings.

[51] Choi J.W., Cho H.M., Kwak E.K., Kwon T.G., Ryoo H.M., Jeong Y.K., Oh K.S., Shin H.I. (2004). Effect of Ag-Doped Hydroxyapatite as a Bone Filler for Inflamed Bone Defects. Key Eng. Mater..

[52] Levengood S.K.L., Zhang M. (2014). Chitosan-based scaffolds for bone tissue engineering. J. Mater. Chem. B.

[53] Bagheri-Khoulenjani S., Taghizadeh S.M., Mirzadeh H. (2009). An investigation on the short-term biodegradability of chitosan with various molecular weights and degrees of deacetylation. Carbohydr. Polym..

[54] Thein-Han W.W., Misra R.D.K. (2009). Biomimetic chitosan-nanohydroxyapatite composite scaffolds for bone tissue engineering. Acta Biomater..

[55] Ishida T. (2018). Antibacterial mechanism of Ag+ ions for bacteriolyses of bacterial cell walls via peptidoglycan autolysins, and DNA damages. MOJ Toxicol..

[56] Feng Q.L., Wu J., Chen G.Q., Cui F.Z., Kim T.N., Kim J.O. (2000). A mechanistic study of the antibacterial effect of silver ions on *Escherichia coli* and *Staphylococcus aureus*. J. Biomed. Mater. Res..

[57] Lozoya-Rodríguez D.A., De Lima R., Fraceto L.F., Ledezma Pérez A., Bazaldua Domínguez M., Gómez Batres R., Reyes Rojas A., Orozco Carmona V. (2017). Development of HA/Ag-NPs Composite Coating from Green Process for Hip Applications. Molecules.

[58] Fenech M. (2007). Cytokinesis-block micronucleus cytome assay. Nat. Protoc..

[59] Ruggiero R., Conte R., Marano R.M., Serratore G., Aiello E., Facente A., Ambrogio G., Tatullo M., Paduano F. (2025). Single point incremental forming of Ti-6Al-4V titanium alloy for biomedical applications: Process optimization and in vitro biocompatibility assessment. J. Mech. Behav. Biomed. Mater..

[60] Piszczek P., Radtke A., Ehlert M., Jędrzejewski T., Sznarkowska A., Sadowska B., Bartmański M., Erdoğan Y.K., Ercan B., Jedrzejczyk W. (2020). Comprehensive Evaluation of the Biological Properties of Surface-Modified Titanium Alloy Implants. J. Clin. Med..

[61] Jena P., Mohanty S., Mallick R., Jacob B., Sonawane A. (2012). Toxicity and antibacterial assessment of chitosan-coated silver nanoparticles on human pathogens and macrophage cells. Int. J. Nanomed..

