# Supplementary material for: Bacterial Adhesion on Orthopedic Titanium Implants Coated with Silver-Doped Hydroxyapatite and Chitosan
Source: Molecules. 2026 Oct 4;31(19):3537. doi: 10.3390/molecules31193537 (PMC13667199; doi:10.3390/molecules31193537)
Supplement: Supplementary file 1 [file molecules-31-03537-s001.zip › molecules-4537685-supplementary.pdf]

Bacterial Adhesion on Orthopedic Titanium Implants Coated with Silver-Doped Hydroxyapatite and Chitosan

AM. Zore[a], N. Matjaković Mlinarić[a], A. Abram[b], R. Štukelj[a], M. Modic[b], A. Leskovac[c], S. Petrović[c], V. Stanić[c], S. Ghannouchi[d], R. Dahmane[a], K. Bohinc[a]

[a] Faculty of Health Sciences, University of Ljubljana, Ljubljana, Slovenia

[b] Jozef Stefan Institute, Ljubljana, Slovenia

[c] Vinča Institute of Nuclear Sciences- National Institute of the Republic of Serbia, University of Belgrade, Belgrade, Serbia

[d] University of Sousse, Faculty of Medicine, Institute of Anatomy, Sousse, Tunisia

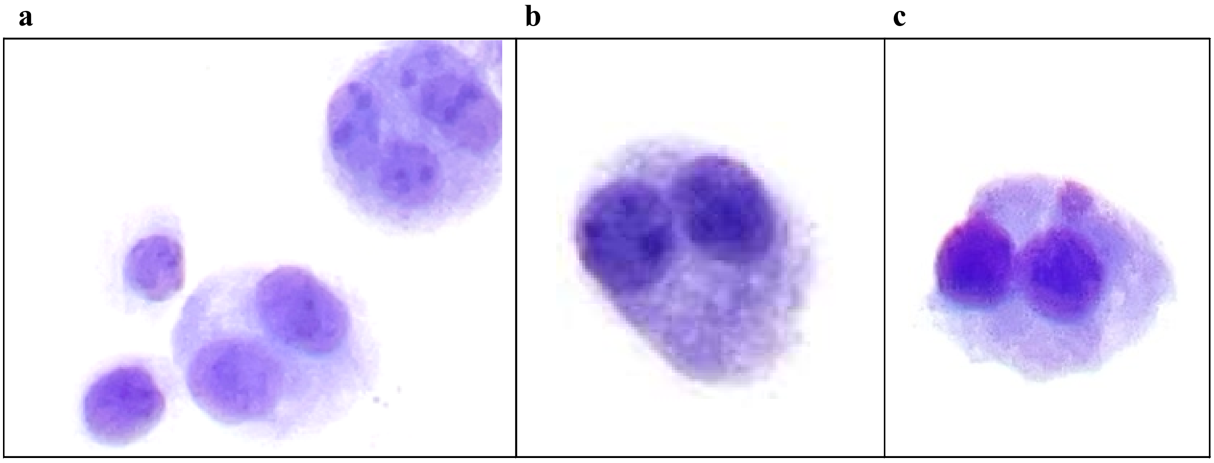

Figure S1. Micrographs showing mononucleated, binucleated and polynucleated cells (a), normal binucleated cell (b), and binucleated cell with micronucleus (c) following Giemsa staining.

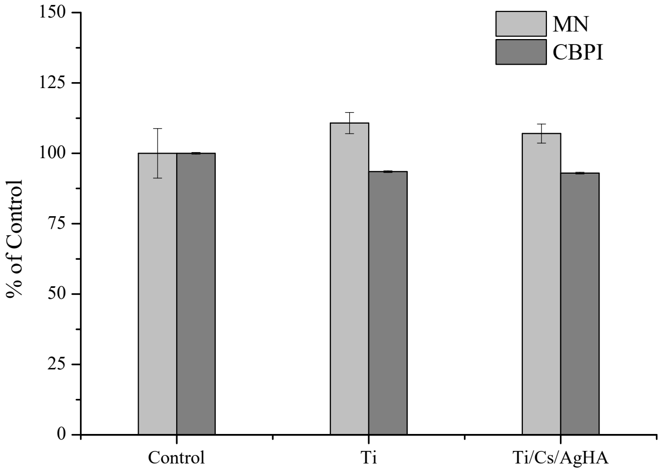

Figure S2. Incidence of micronuclei (mean±SD) and cytokinesis-block proliferation index (mean±SD) in lymphocytes exposed to the Ti and Ti/CS/AgHA. The values are expressed as a percentage of control, set to 100%.
